# Generating political priority for breastfeeding and the adoption of Kenya’s 2012 BMS act: the importance of women’s leadership

**DOI:** 10.1186/s12992-025-01127-2

**Published:** 2025-05-29

**Authors:** Maryanne Wamahiu, Phillip Baker, Tim Dorlach

**Affiliations:** 1 Faculty of Life Sciences: Food, Nutrition and Health, University of Bayreuth, Kulmbach, Germany; 2 Bayreuth International Graduate School of African Studies, University of Bayreuth, Bayreuth, Germany; 3 School of Public Health, University of Sydney, Sydney, Australia

**Keywords:** Breastfeeding, Breast milk substitutes, Formula milk, International code, Kenya, Male allies, Women’s political leadership, Transnational corporations

## Abstract

**Background:**

The World Health Organization recommends initiating breastfeeding in the first hour of life, exclusive breastfeeding for six months, and continued breastfeeding for at least two years. Aggressive marketing of breast milk substitutes (BMS) undermines breastfeeding and is linked to adverse child and maternal health outcomes. This is particularly problematic in the Global South, where socioeconomic conditions often amplify the risks associated with BMS. The adoption of national BMS legislation in line with the 1981 International Code of Marketing of Breast-milk Substitutes is therefore crucial but difficult due to strong opposition from the transnational formula milk industry. Breastfeeding advocates in Kenya were able to overcome this powerful opposition when the country adopted a strict BMS Act in 2012, which has since facilitated and protected remarkable improvements in breastfeeding rates. We conduct a qualitative case study to identify the political enablers of the successful adoption of this important law.

**Results:**

BMS legislation was first politically debated in Kenya in the 1980s following mobilization of women-led civil society organizations, namely the Breastfeeding Information Group and the Maendeleo ya Wanawake Organization. The issue re-emerged on the political agenda in the 2000s but faced opposition from the transnational formula milk industry. Kenya’s BMS Act was ultimately adopted during a policy window opened by a constitutional reform. Support for the adoption of this landmark law was led by effective female political leaders, including public health minister Beth Mugo, the ministry’s nutrition division head Terrie Wefwafwa, and members of the Kenya Women’s Parliamentary Association. In the formulation and adoption of the law, these female leaders received important support from international organizations, such as the United Nations Children’s Fund, as well as from powerful male allies, including president Mwai Kibaki.

**Conclusions:**

The Kenyan case illustrates how women’s political leadership can counteract the power of the transnational formula milk industry and help achieve strict BMS legislation. Effective female leadership for BMS legislation can occur in various political offices and positions, including those of ministers, legislators and bureaucrats. Female leaders can leverage their own influence by strategically exploiting policy windows and recruiting male allies.

**Supplementary Information:**

The online version contains supplementary material available at 10.1186/s12992-025-01127-2.

## Introduction

Breastfeeding is considered the best feeding for infants. Breastfeeding reduces the likelihood of common childhood illnesses and malnutrition [1–3], women’s risk of diabetes and reproductive cancers [4–6] and has significant benefits for sustainability, climate change mitigation, and food security of the child [7, 8]. In contrast, bottle feeding with breast milk substitutes (BMS) can pose severe risks to infant health, especially in the Global South. Contamination from unsafe water can cause diarrhea, while over-dilution of formula can result in undernutrition [1, 2]. The World Health Organization (WHO) therefore recommends initiation of breastfeeding within one hour of birth, exclusive breastfeeding for six months, and continued breastfeeding up to at least two years of age [9]. While important progress has been made in recent years, only 48% of the world’s babies are exclusively breastfed [10].

One of the main reasons behind low breastfeeding rates is the aggressive marketing of BMS by the transnational formula milk industry. These marketing practices include misleading advertising on the supposed superiority of BMS to breast milk, offering free samples to mothers in hospitals, and promotion through health workers [11–13]. While BMS can fulfil important functions, for instance, when breastfeeding is medically contra-indicated in rare cases or to support women’s labor force participation, aggressive marketing practices can effectively prevent women from making informed decisions.

BMS marketing became a globally salient political issue in the 1970s, e.g., through the 1974 report “The Baby Killer”, the 1975 documentary film “Bottle Babies”, and the 1977 launch of a consumer boycott against Nestlé [14]. In 1981, the World Health Assembly (WHA) passed the International Code of Marketing of Breast-milk Substitutes [15–17]. This International Code is a “set of recommendations to regulate the marketing of breast-milk substitutes, feeding bottles and teats”, which “aims to stop the aggressive and inappropriate marketing of breast-milk substitutes” [18]. The International Code is a living document, which is updated biannually through WHA resolutions, that have the same legal status as the initial code, in response to WHO technical guidance and evolving industry practices. When we refer to the International Code, we therefore mean the code and subsequent resolutions.

While the International Code represents a landmark achievement in the global regulation of BMS marketing, it was adopted as a non-binding code of conduct [15, 17, 19]. The adoption of binding national legislation in line with the International Code is therefore crucial. Yet, by 2022, only 32 countries worldwide had adopted legislation that it “substantially aligned” with the International Code [20]. The existing literature has already identified various enabling factors of programs and policies to protect, promote and support breastfeeding, including “evidence-based advocacy” and “political commitment” [21–26]. Other studies have focused on the barriers to the adoption of strict BMS legislation, in particular extensive corporate lobbying, or corporate political activity, by the transnational formula milk industry [27–30]. As corporate lobbying clearly presents a major barrier to the adoption of strict BMS legislation, more fine-grained research is needed on the political factors and strategies that can enable the adoption of strict BMS legislation at the national level.

To contribute to a better understanding of the political enablers of strict BMS legislation, we conduct a case study of Kenya, which adopted the Breast Milk Substitutes Act (BMS Act) in 2012. The Act has been praised as an important piece of legislation, not only for the protection, promotion and support for breastfeeding in Kenya, but also for the improvement of nutrition and health outcomes [31]. However, little is known about the political factors that made its enactment possible [15]. In this paper, we analyze the policy process that led to the successful adoption of Kenya’s 2012 BMS Act.

## Research design

We conduct a case study of Kenya’s 2012 BMS Act. This law introduced several important policy changes, most importantly the prohibition of all advertising and promotion of BMS. BMS covered by the Act include infant formula for infants below 6 months, follow-on formula for children between 6 and 24 months and complementary foods. Follow-on formula is among the fastest growing segments of the global baby food market but is criticized for contributing to child obesity (as it often contains added sugar, especially in low- and middle-income countries) and for facilitating so-called “cross-promotion” of breast milk substitutes, a strategy used to circumvent restrictions on infant-formula marketing [13, 32, 33]. The law also established a National Committee on Infant and Young Child Nutrition, which fulfils a policy advisory role to the Minister for Health.

**Fig. 1:**
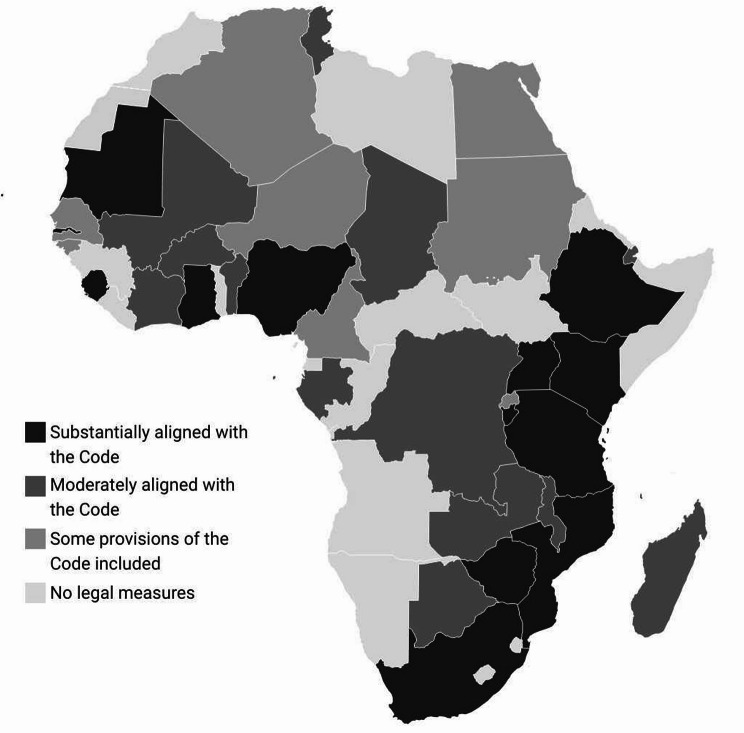
National legal status on the International Code in Africa, 2022 **Source**: WHO (2022) [20]

**Fig. 2:**
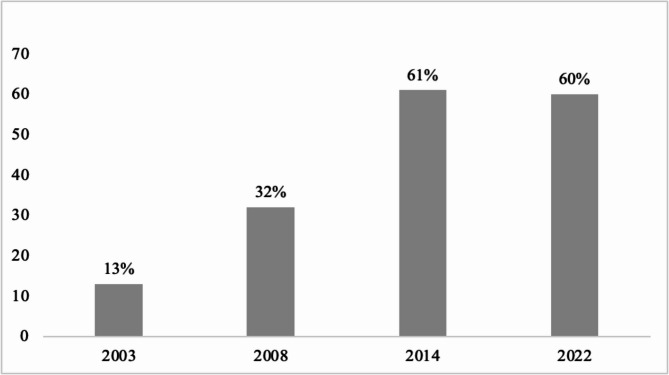
Exclusive breastfeeding (0–5 months) trends in Kenya Source: Kenya Demographic and Health Surveys (2003, 2008, 2014, 2022) [35–38]

In 2022, Kenya was one of 14 African countries with legislation substantially aligned with the International Code (Fig. 1). Overall, Kenya ranked fifth in Africa in terms of International Code alignment (with 82 out of 100 possible points), trailing only Sierra Leone (99), South Africa (87), Ethiopia (85) and Nigeria (84) [20]. Kenya has recorded steady improvements in exclusive breastfeeding rates since the early 2000s (Fig. 2), with the highest rise recorded between 2008 (32%) and 2014 (61%). Together with other reforms, such as the expansion of paid maternity leave in 2007 [34], the 2012 BMS Act has been key in facilitating and protecting these improvements.

Physical archival research and in-person interviews were conducted during three rounds of fieldwork in Nairobi, Kenya, between 2023 and 2024. Primary documents reviewed for this study include archival documents accessed in the Kenya National Archives, including correspondence, newsletters and minutes from files annexed under the Ministry of Health Nutrition Division, Kenya Bureau of Standards, and the Ministry of Social Services. The Parliamentary Hansard and regulatory documents were also reviewed, some online and others in the Kenya Parliamentary Library. News coverage was accessed from the Nation Media and Standard Group libraries, in addition to online repositories. 21 semi-structured key-informant interviews were conducted, both in-person and remotely, with representatives from relevant government institutions, civil society organizations and academia (see Table S1). Interviewees were selected through purposive snowballing [39].

To develop an explanation of the successful adoption of Kenya’s 2012 BMS Act, we used inductive process tracing, a method that is ideally suited for studying the causes of specific policy outcomes [40, 41]. Our process tracing analysis is complemented by a within-case comparison [42] of the successful adoption of the 2012 BMS Act with the failed introduction of BMS legislation in the early 1980s. We employ qualitative content analysis [43] to extract relevant information from our interview and document sources.

### Theoretical framework

Our case study is guided by the policy cycle framework of the policy process [44]. This framework “has become the conventional way to describe the chronology of a policy process” [44]. It divides the policy process into a sequence of distinct stages, most commonly agenda setting as well as policy formulation, adoption, implementation and evaluation. We find the policy cycle framework particularly useful for studying the politics of BMS regulation, as it allows us to distinguish the political dynamics of the struggle for strict BMS regulation during these different phases [23, 24]. In this paper, we focus on the agenda-setting, formulation and decision-making processes that led to the adoption of Kenya’s 2012 BMS Act. Here we also draw on the literature on the determinants of political commitment and priority for nutrition [45, 46].

In substantive theoretical terms, we draw on the literature on women in politics [47]. By studying what can be described as “women’s political leadership” [48], this literature has highlighted how women can bring about policy change when achieving positions of power, be it in the executive, legislature or judiciary. In other words, this literature investigates when and how descriptive representation, i.e., women’s presence in leadership positions, also results in substantive representation, i.e., representation of women’s interests [49]. Studies on African countries, where elected female heads of state have remained relatively rare, have mostly examined the policy impact of female legislators and cabinet members [49–51]. This literature has highlighted the importance of cross-party women’s caucuses and the strategic recruitment of “male allies” in enhancing female political leaders’ ability to bring about policy change [52, 53].

We also draw extensively on the theoretical literature on the transnationalization of national policy making [54]. Research has demonstrated that domestic policymaking processes, especially but not only in the Global South, are often strongly influenced by transnational actors and networks. Accordingly, domestic policy entrepreneurs are often embedded in “transnational epistemic communities” [55] or “transnational advocacy networks” [56]. Likewise, transnational corporations often use their immense economic resources and political power to influence domestic policy decisions [57, 58]. The policy field of BMS regulation is characterized by intense transnationalization. National BMS regulations are regularly opposed by transnational formula milk industry [28, 59]. At the same time, national advocates for stricter BMS regulation are usually supported by a transnational network that includes the international organizations UNICEF and WHO as well as the non-governmental International Baby Food Action Network (IBFAN) [19, 28, 59]. Indeed, the case of IBFAN directly informed the original development of the theoretical concept of “transnational advocacy networks” [16, 56].

## Results

The adoption of Kenya’s 2012 BMS Act was the culmination of decades-long political efforts for BMS legislation. In the following sections, we causally reconstruct the political process that led to the successful adoption of the Act. We begin with a brief account of a first attempt to introduce BMS legislation in the 1980s. We then focus on the re-emergence of BMS legislation on the policy agenda in the 2000s and the parliamentary debate and adoption of the law in 2012. In a nutshell, we argue that the efforts of women leaders, supported by male allies and transnational networks made a strong contribution towards the adoption of this landmark BMS law.

### Early problem definition and advocacy

Like in many other countries, breastfeeding and BMS marketing first began being politically problematized in Kenya during the 1970s. Transnational BMS producers had first entered the Kenyan market in 1913 [60]. By the 1970s, BMS marketing had become widespread and aggressive. Nestlé’s Lactogen was the most advertised brand on Swahili radio at the time, alone accounting for 11% of all (not just BMS) advertisements [61]. Rampant advertising of BMS was associated with low exclusive breastfeeding rates and what Derrick and Patrice Jelliffe have described as “commerciogenic malnutrition” [62]. Indeed, the problem was so pronounced that Kenyans reportedly developed the term *chupa-itis* to “describe diarrhea and dysentery in bottle-fed babies (chupa means bottle in Kiswahili)” [63]. The situation in Kenya became a central reference point in international debates about the specific risks of BMS in the Global South. For instance, the influential 1975 documentary film “Bottle Babies” by West German filmmaker Peter Krieg, which became instrumental in the 1977 Nestlé boycott, was primarily shot in Nairobi’s Kenyatta National Hospital [64].

Women-led civil society organizations, supported by international donors, were central in advocating for stricter regulation of BMS marketing in Kenya. The first among these was the small, Breastfeeding Information Group (BIG), a voluntary women-led organization focused on breastfeeding promotion. The organization was established in 1978 in Kenya, as the first African breastfeeding advocacy group by the Ugandan Margaret Kyenkya and a group of women from various countries including Rachel Musoke from Kenya [65, 66]. BIG’s establishment was motivated by an encounter of Margaret Kyenkya with the American Helen Armstrong, a former Peace Corps and La Leche League volunteer [65, 67] and was also supported by other expatriate volunteers, such as the Canadian Linda George [68]. BIG became affiliated with IBFAN, which was founded in 1979. In the late 1980s, BIG employed twelve salaried staff members and had received most of its funds from international donors, including Norwegian International Aid for Development (NORAD), Oxfam, UNICEF, the Danish International Development Agency and the Ford Foundation [69]. Working closely with the Ministry of Health, BIG was able to push the breastfeeding agenda through individual counselling and talks with mothers in maternal and child health facilities, providing in-service training in breastfeeding management to health workers and women’s groups in rural areas, and conducting research on breastfeeding trends in the 1980s and early 1990s [70].

In 1982, BIG successfully recruited an important ally in its fight to promote breastfeeding and push for strong national BMS regulation, namely the *Maendeleo ya Wanawake Organization* (MYWO), then Kenya’s “biggest and politically powerful grassroots women’s organization” [65]. At the time, MYWO largely relied on funding from international donors. For instance, in 1979, MYWO launched a Maternal Child Health and Family Planning program with funding from Pathfinder International, the World Bank, USAID and NORAD [71]. MYWO was particularly influential between 1971 and 1984, when it was chaired by Jane Kiano, who enjoyed strong ties with Kenya’s ruling party and was the spouse to an influential, long-serving minister [65, 72].

### A first attempt at BMS legislation

In the early 1980s, and especially after the adoption of the International Code in 1981, BIG and MYWO jointly advocated for the introduction of strong national BMS legislation in Kenya through a Kenya Code [73].^1^ MYWO’s chairperson, Jane Kiano, threw her political weight behind the cause, reportedly demanding in a meeting that “I want this Code. Definitely. And I want it soon.” [65]. The formulation of this Kenya Code was initially led by the Ministry of Health but was soon taken over by the Kenya Bureau of Standards (KEBS), an agency under the Ministry of Industry [77]. Representatives of the BMS industry and Nestlé in particular “insisted on being full participants in the drafting process” [65] and were in fact overrepresented according to BIG [78]. Indeed, the 1982 draft of the Kenya Code fell substantially short of the standards set out in the International Code and lacked a clear enforcement mechanism [79]. After further revisions and an influential National Workshop in Infant Feeding Practices in April 1983 [80, 81], the Kenya Code for Marketing of Breast Milk Substitutes was eventually adopted as a voluntary KEBS standard in May 1983 [82].

BIG and MYWO therefore ultimately failed at generating enough political support for BMS legislation, unable to overcome the strong opposition of the BMS industry. Yet, even this adoption of a voluntary code can be viewed as a certain policy achievement. By 1991, only three African countries, Kenya, Nigeria and South Africa, had adopted such voluntary codes, while none had adopted comprehensive and legally binding BMS regulations [83]. It also appears that the 1983 Kenya Code was enforced relatively effectively in public hospitals through a series of health ministry directives. In conjunction, the Kenya Code and these directives “effectively banned the free supply of infant formula to Kenyan hospitals” [84]. Yet, the goal of adopting comprehensive BMS legislation remained unattained.

### Declining interest in BMS legislation

This early policy window for adopting BMS legislation closed again after the mid-1980s, as both BIG and MYWO were weakened. Kiano, MYWO’s powerful chairperson, who had embraced the issue of BMS regulation, stepped down in 1984 [71]. Kyenkya, BIG’s founding director, moved to New York in 1987 and started working for UNICEF [65]. These personnel changes were accompanied by a funding crunch for civil society organizations. Oxfam, which had paid Kyenkya’s salary as BIG’s coordinator, withdrew from funding BIG in 1987 [69]. NORAD, which had been a major funder of both BIG and MYWO was expelled from the country in 1990 due to an unrelated political conflict between Kenya and Norway [69, 71]. All of these changes occurred in the context of the increasingly authoritarian presidency of Daniel arap Moi.

In 1989, the issue of BMS legislation briefly flared up again. At a regional conference of IBFAN Africa in Nairobi, Mwai Kibaki, Kenya’s health minister from 1988 to 1991 (who would later become president and sign the 2012 BMS Act into law), expressed commitment to enact a binding Kenya Code, “to be followed by marketers of baby foods or infant formula to protect mothers from being cheated and confused” [85]. However, nothing came out of Kibaki’s pledge at the time, likely because of a fallout with President Moi and a relatively brief tenure as health minister [86].

In the 1990s, the issue of BMS legislation largely disappeared from Kenya’s political agenda. The report of a fact-finding mission, deployed by UNICEF and IBFAN, revealed that, in the second half of the 1990s, “a broad range of actions and funding to assist protection, promotion and support of breastfeeding, have declined very markedly”, while “interest” in binding BMS legislation has “waned” [87]. Some of this had, without doubt, to do with the continued influence of BMS producers and with the fact that KEBS (rather than the health ministry) remained in charge of BMS regulation [87]. Yet, it was also a result of rising rates of HIV infections in Kenya and growing concerns about the risk of HIV transmission through breastfeeding [88]. In 1997, this led to a specific recommendation by the Kenyan health ministry that “women with HIV will be advised to avoid breast-feeding their children and use alternative feeds” [88]. In sum, political priority for BMS regulation had reached a low point in the late 1990s.

### Renewed advocacy and an opening policy window

Advocacy for BMS legislation began to re-emerge in the early 2000s. One specific transnational event that “renewed interest” [87] was the participation of three Kenya-based breastfeeding advocates in a training course on International Code compliance, hosted by IBFAN’s International Code Documentation Centre (ICDC) in Penang in September 2000. The ICDC was established by IBFAN in 1985 “to conduct courses on Code implementation and Code monitoring” [12, 89]. The three participants from Kenya included Rachel Musoke, a pediatrics professor and co-founder of BIG, Catherine Muyeka Mumma, a lawyer from the Office of the Attorney General, as well as a UNICEF program officer [87]. After the training course, there was “some resolve to move the Code from the Bureau of Standards to the Ministry of Health” and Mumma was “highly motivated to draft new legislation once she gets the go ahead” [87]. Mumma subsequently began working on a draft law, which would later form the basis for the 2012 BMS Act [90].

The policy window for BMS legislation slowly began to open with the election of President Mwai Kibaki in December 2002, which ended the 24-year rule of Daniel arap Moi. From the beginning, Kibaki demonstrated a greater willingness to support women’s political leadership. His appointment of Charity Ngilu as the first woman Minister of Health (2003–2007) indicated his trust in women’s leadership in the health sector. Appointing “a woman at the helm of health was a strategic decision that served to refocus the ministry to women’s healthcare, which was in dire need of attention” [91]. In 2007, the publication of a new South African study on the effects of exclusive breastfeeding on mother-to-child HIV transmission motivated the health ministry leadership to revisit the issue of BMS regulation [92]. This resulted in the formation of a technical team within the health ministry, headed by Annah Wamae of the Division of Child and Adolescent Health, that began to reconsider the issue of BMS regulation.

The policy window further opened after the highly disputed general election of December 2007, in which both Kibaki and opposition leader Raila Odinga had claimed victory. After post-election violence that claimed over 1,000 lives, the two conflicting parties entered into a power sharing agreement that resulted in a “grand coalition” government with Kibaki as president and Odinga as prime minister from 2008 to 2013. This arrangement shaped the political process of Kenya’s 2012 BMS Act in several ways. It facilitated an important constitutional reform in 2010 and translated into a less divided parliament [93], which would diminish the role of party lines in the legislative adoption process of the law in 2012.

### Creation of a public health ministry

Most immediately, the formation of a coalition government in 2008 had important consequences for Kenya’s health ministry, which was back in charge of BMS legislation. The power sharing agreement led to the formation of an inflated, 43-person cabinet [94]. To create new cabinet positions, some ministries were split up during the 2008–2013 period. This included the Ministry of Health, which was divided into the Ministry of Medical Services (MoMS) and the Ministry of Public Health and Sanitation (MoPHS). This newly created MoPHS sought to shift the “focus of government and donor spending from curative to preventive health strategies” [95]. The Division of Child and Adolescent Health, which had begun to re-engage with the issue of BMS regulation in 2007, was allocated to the MoPHS. Significantly, the MoPHS and the MoMS soon displayed distinct perspectives on BMS. The MoMS viewed BMS primarily as a curative medical product. The MoPHS, in contrast, viewed BMS primarily as a hindrance to breastfeeding, especially in the many cases where formula feeding was not necessary. The creation of the MoPHS therefore provided a distinct institutional space for this public health perspective on BMS.

President Kibaki appointed Beth Mugo to lead the new MoPHS. Mugo would become the most influential political actor behind the 2012 BMS Act. At the time, she was a member of Kibaki’s Party of National Unity and had served as an Assistant Minister for Tourism and Information in Kibaki’s previous government [96]. Mugo was well connected in Kenya’s political elite, in part because she was the “favorite niece” of Jomo Kenyatta, Kenya’s first post-independence president [97], and enjoyed a close relationship with President Kibaki [96]. Mugo had been a member of Kenya’s (then) unicameral parliament since 1997. Doubling as minister and legislator from 2008 to 2013 allowed Mugo to be closely involved in both the formulation and adoption of the BMS Act. Beth Mugo was broadly committed to public health promotion, for instance, through the implementation of Kenya’s 2007 Tobacco Control Act [98, 99].

By the time Beth Mugo became Minister of Public Health and Sanitation, she was already a key figure in the Kenyan women’s movement. Mugo ran a family business and was Kenya’s national chairperson of the International Federation of Business and Professional Women from 1984 to 1987 [99]. When Mugo became a member of parliament (MP) in 1997, she was one of six elected women MPs [100]. In 2000, Mugo sponsored a landmark affirmative action bill that sought to increase the political representation of women and other marginalized groups [101]. In 2001, Mugo co-founded (together with Martha Karua and Charity Ngilu) the Kenya Women’s Parliamentary Association (KEWOPA), a bi-partisan women’s caucus dedicated to supporting women’s representation and women-centered policies in parliament [102]. KEWOPA would later play a vital role in the parliamentary adoption of the BMS Act.

### Creation of a nutrition division

The new Ministry of Public Health and Sanitation created a dedicated new Division of Nutrition, focusing on the implementation of preventative nutrition interventions. This portfolio was previously with a Unit, which had more limited autonomy. Initially, the allocation of nutrition-related departments was contested between the MoPHS and the MoMS [103]. The Division of Nutrition was eventually allocated to the MoPHS (while a new Unit of Clinical Nutrition was created in the MoMS). From 2008 to 2014, this division was headed by Terrie Wefwafwa, a nutritionist and career civil servant, who had worked as a provincial nutrition officer since the 1970s. Her strong support for BMS regulation was informed by her experience with the negative effects of bottle feeding as a District Nutrition Officer. During her time at the helm of the Division of Nutrition, Wefwafwa showed strong political leadership for public health nutrition, ranging from breastfeeding promotion to mandatory food fortification [104].

Wefwafwa’s work emphasized better coordination among stakeholders and a strengthening of government leadership in the field of nutrition. Historically, nutrition programming in Kenya was often fragmented and dominated by NGOs [105]. After a severe drought and famine in 2006 [106] and the creation of the Division of Nutrition in 2008, development partners such as UNICEF and the WFP began clarifying their roles and responsibilities. This resulted in a 2009 “partnership framework”, which formalized a “common vision for nutrition services”, including the “transition of leadership and governance” to the health ministry [105]. In practice, this resulted in the creation of an interagency coordination committee and several government-led working groups, including a Maternal, Infant and Young Child Nutrition (MIYCN) Technical Working Group [105]. The MIYCN working group, which brought together representatives from the government, WHO, UNICEF, IBFAN and other NGOs, played an important role in the formulation of the 2012 BMS Act.

The new political leadership at the Ministry of Public Health and Sanitation soon focused on Kenya’s poor trends in child mortality indicators. The country’s infant mortality rate, after years of decline, had actually increased in the 1990s to reach 77 deaths per 1,000 live births in 2003 [35]. While this rate was back down to 52 in 2008/09, this was still significantly higher than the Kenya Vision 2030 target of 25 by 2012 [37, 107]. The MoPHS and its partners argued that the promotion of exclusive breastfeeding during the first six months, even for mothers with HIV, was one of the most effective interventions for reducing child mortality [105, 108]. And, crucially, they re-focused on the regulation of BMS marketing as a central tool for promoting and protecting exclusive breastfeeding. In this context, IBFAN Africa’s 2007 regional conference in Maputo was an impactful event, as it highlighted that Kenya had become a regional laggard in breastfeeding protection and International Code implementation. Indeed, Tanzania and Uganda, two of Kenya’s key neighboring countries, already had BMS legislation in place by 2006 [93].

### Drafting of the BMS bill

The main drafting process of Kenya’s BMS Act began in 2008 under the leadership of Terrie Wefwafwa [109]. This process was supported by the assignment of a state legal officer from the Attorney General’s office. The drafting process occurred in close coordination with WHO and UNICEF, in particular with David Clark, a legal specialist from UNICEF headquarters in New York [110]. The first complete draft of Kenya’s BMS Act was completed by 2012 and was largely in line with the International Code.

The policy window for introducing the BMS draft law into parliament fully opened in August 2010 when Kenya adopted a new constitution through a referendum, replacing the country’s previous constitution of 1969. Just two weeks after this referendum, Beth Mugo publicly announced that “plans are underway to enact the regulatory law of breast milk substitutes on the Kenyan market” [111]. The new constitution facilitated the adoption of BMS legislation in multiple ways. It enshrined human rights through a comprehensive bill of rights, which in the following years gave rise to a general atmosphere of reform to realize these newly defined rights [112]. New constitutional provisions that “every child has the right to […] basic nutrition, shelter and health care” as well as to “parental care and protection” provided a direct justification for the BMS Act [113].

The new constitution also cleared a crucial veto point that had previously hindered stricter BMS regulation. BMS legislation had previously been stalled by Kenya’s long-time attorney general Amos Wako, who had been in office since 1991. He had acquired a reputation for being complicit in various corruption cases [91]. As “the principal legal adviser to the Government” [113], the Attorney General plays a central role in Kenya’s legislative process, including in legislative drafting. Indeed, Beth Mugo would later report that a draft bill once even disappeared from the Attorney General’s office [114, 115]. Kenya’s new constitution, however, explicitly required Wako to step down within one year, which he did in August 2011 [116]. Wako was succeeded by the politically much less entrenched Githu Muigai, which cleared the crucial veto point of the Attorney General’s office.

### Parliamentary debate of the BMS bill

On 2 August 2012, the Breast Milk Substitutes (Regulation and Control) Bill was introduced into Kenya’s (then still unicameral) National Assembly [117]. The bill was guided through the parliamentary process by Beth Mugo, who was also a member of parliament. Mugo was motivated to get the bill passed quickly, as a breast cancer diagnosis in January 2012 made her consider retirement from political office, which she would later announce in December 2012 [118].

Throughout the parliamentary process, the bill faced strong opposition from the transnational formula milk industry. While BMS producers abstained from speaking out openly against the bill, they strongly mobilized against the bill behind the scenes. Farah Maalim, the deputy speaker of parliament and one of Mugo’s major allies in support of the BMS bill, noted during the parliamentary debate that “it is an open secret that the stakeholders from the industry have been camping out there” [119]. Maalim later specified that “when we were enacting the breast milk law […] Nestle gave us a serious pushback. The Nestle lobby in Parliament was well oiled & very loud” [120]. Incidentally, Nestlé had replaced its Nairobi-based regional Chief Executive Officer in June 2012 “to attain a firmer grip on the [African] region” [121]. The Kenya Association of Manufacturers (KAM) opposed the law explicitly, as it would “hinder the business of affected companies” [122].

The main parliamentary debate of the BMS bill occurred on 19 and 20 September 2012. Representatives of UNICEF initially followed the debate from parliament’s public gallery but later decided to leave to avoid the impression of an externally imposed law. While no member of parliament openly argued against the bill as such, there was strong pressure to weaken the bill through amendments. In particular, three (ultimately rejected) amendments would have significantly reduced the stringency of the BMS bill. A first amendment (proposed by Charles Keter) would have added three industry representatives, nominated by the Kenya Association of Manufacturers (KAM), to the newly created National Committee on Infant and Young Child Feeding, to ensure that “the manufacturers have their representation” [119]. This would have granted industry representatives a privileged position to influence the implementation, monitoring and enforcement of the law. A second amendment (also proposed by Keter) would have allowed enforcement officers to access the premises of BMS manufacturers or distributors only with a court warrant, which would have greatly complicated enforcement. A third amendment (proposed by Robert Monda) would have allowed health workers and proprietors to accept financial assistance from the formula milk industry, e.g., through research grants, as long as there was full disclosure. This amendment would have arguably gone furthest in weakening the bill, as it would have opened substantial space for conflict of interest and industry influence over the healthcare system. the promotion of BMS and complementary feeding products. All three of these amendments were eventually rejected and the BMS bill was passed with only minor amendments on 20 September 2012.

### Sources of support for the BMS bill

The first and most central factor behind the successful adoption of the BMS bill was the strong, bipartisan support by all women MPs. This support was institutionally facilitated by KEWOPA, which endorsed the bill [123]. KEWOPA members embraced the bill as a women-centered and women-led law. For instance, Rachel Shebesh argued that such a bill was only possible “because we have a woman as a Minister and that is why we need more women Ministers” [119]. During the parliamentary debate, no female MP opposed the bill.^2^ Instead, many KEWOPA members actively supported it. Key among those was Martha Karua, who together with Beth Mugo had co-founded KEWOPA in 2002 and had served as justice minister from 2005 to 2009. Karua’s support was significant as she was the former Minister for Justice and Constitutional Affairs, often described as the “iron lady” of Kenyan politics. On several occasions, Karua rose to support Mugo in defending the bill against hostile amendments.

While KEWOPA’s support was central in establishing a bipartisan basis of support, female MPs made up only 10% of MPs in 2012 [124]. Hence, broad support from “male allies” was essential for the adoption of the bill [53]. Such male support was not a given, as all parliamentary opposition to the bill was voiced by male MPs (see Table S2). Indeed, Karua had explicitly called out her “male colleagues” for attempting to delay the parliamentary debate on the bill [114]. But Mugo managed to successfully recruit several influential male legislators to support the bill. After Mugo had introduced the bill on 19 September, she was seconded by Amos Kimunya, the male transport minister. Indeed, the fact that Mugo decided to have her bill co-sponsored by a male rather than a female legislator even surprised the parliament’s deputy speaker in charge of the session, who noted to Mugo that “my presumption was that you would look for a lady who has breastfed like you” [119]. The bill was then formally supported by two other male MPs before other female MPs expressed their support. Another important male ally was trade minister Moses Wetangula, whose support was significant given that the trade ministry often sides with industry interests.

### Overcoming opposition to the BMS bill

Another factor that contributed to the successful adoption of strict BMS legislation was the effective bypassing of public participation requirements. Public participation requirements, e.g., in the form of “notice and comment” procedures, are often introduced as an instrument to “democratize” policymaking. In practice, however, formalized public participation requirements have been shown to create an access point to policymakers that is primarily used by business actors [125]. Kenya’s 2010 constitution introduced the requirement that parliament “facilitate public participation and involvement in the legislative and other business of Parliament and its committees” [126]. In 2012, however, the modalities of such public participation in the legislative process had not yet been clarified [126]. The parliamentary proponents of the BMS bill exploited this temporary opportunity and proceeded without formal public participation in order to limit industry influence. The Kenya Association of Manufacturers later lamented the limited involvement of industry actors in the formulation of the law and the deliberate exclusion of the private sector from the newly established National Committee on Infant and Young Child Feeding, which was to advise the government on the implementation of the law [122, 127, 128]. This strategic bypassing of public participation mechanisms limited industry influence and thus contributed to the successful adoption of the BMS Act.

In October 2012, after the BMS bill had already been passed by parliament, the Global Alliance for Improved Nutrition (GAIN) sought to convince the government to not sign it into law [129]. GAIN is a public-private partnership focused on reducing micronutrient deficiencies through food fortification [130]. At the time, GAIN was primarily funded by the Bill & Melinda Gates Foundation, while its business partner network included the transnational formula milk corporations Danone and DSM [129, 131]. GAIN prepared an antagonistic policy briefing that it sent to the MoPHS [129]. GAIN’s main concern was that the bill also sought to restrict the advertising of “complementary food products”. But GAIN did not just advocate for the exemption of specific, relatively more accepted complementary food products, such micronutrient powders for home fortification, which reportedly was negotiated anyway [132]. Instead, GAIN lobbied for weaker regulation of “all complementary foods” [129], which would have also included growing-up (or toddler) formula. To convince the government not to proceed, GAIN claimed that the bill was “not currently consistent with international recommendations or Scaling Up Nutrition (SUN) interventions for complementary feeding” and suggested that “if this Act is adopted, there is a risk of reduction of investment in infant and young child nutrition” [129].^3^ Kenya’s government, however, was unmoved by this lobbying attempt and President Kibaki signed the BMS bill into law on 11 October 2012.

## Discussion

Our analysis reveals four main political enablers of strict BMS legislation in Kenya: women’s political leadership, the recruitment of influential male allies, support from a transnational advocacy network, and the opening of a policy window. In combination, these four enabling factors allowed advocates of BMS legislation to overcome the power of the transnational formula milk industry.

First, we find that women’s political leadership was central in generating political priority for BMS legislation. The issue was first brought onto Kenya’s political agenda in the 1980s by the women-led civil society organizations BIG and MYWO. The formulation and adoption of the BMS Act in the later 2000s and early 2010s was led by Beth Mugo and Terrie Wefwafwa at the Ministry of Public Health and Sanitation. The support of the bipartisan women’s parliamentary caucus KEWOPA was central in achieving parliamentary approval, even though female MPs made up only 10% of parliament. This is in line with previous research asserting that women’s political leadership can be influential despite limited numerical strength [101]. Overall, our analysis confirms that women’s representation in powerful political positions can significantly contribute toward improved public health [135]. Our findings about the centrality of women’s political leadership for effective BMS legislation mirrors much earlier observations by Derrick and Patrice Jelliffe on the importance of voluntary women’s groups, such as La Leche League, in breastfeeding promotion [62].

Second, we highlight the crucial role of male allies in the adoption of BMS legislation. President Mwai Kibaki was a particularly important ally, who paved the way for women-led health policy by appointing Charity Ngilu as health minister in 2003 and Beth Mugo as public health minister in 2008. Kibaki also provided the ultimate support for BMS legislation by signing the 2012 BMS bill into law. Male allies were also critical in the parliamentary approval process, as women MPs were in the clear minority in the National Assembly. The backing of several of Mugo’s male cabinet colleagues, including Amos Kimunya and Moses Wetangula, was key in generating sufficient parliamentary support for the BMS bill. These findings confirm the importance of male allies for the adoption of women-led laws [52, 53].

Third, we identify transnational advocacy networks as important in supporting government efforts toward strict BMS legislation. Transnational advocacy by IBFAN and training courses offered by the ICDC in Penang contributed to bringing BMS legislation back onto Kenya’s political agenda in the early 2000s. Technical support from international organizations, in particular UNICEF and WHO, aided the formulation of an effective BMS bill. These findings demonstrate that transnational advocacy networks have not only been important in the adoption of the International Code and subsequent resolutions [16, 19] but are also significant in supporting the formulation and adoption of BMS legislation at the national level.

Fourth, our analysis highlights the importance of policy windows, or windows of opportunity, for the adoption of BMS legislation. While women’s political leadership together with support from male allies and transnational advocacy networks were crucial for the formulation and adoption of Kenya’s BMS Act, this law could arguably not have been passed without the opening of a policy window between 2011 and 2013. Key elements of this policy window were the presidency of Mwai Kibaki (2002–2013), the temporary establishment of a dedicated Ministry of Public Health and Sanitation (2008–2013), and the adoption of a new constitution (in 2010) that necessitated the replacement of Kenya’s long-time attorney general (in 2011). These contextual factors explain why the BMS Act was adopted only in 2012. This finding confirms previous research on the centrality of policy windows in other areas of public health nutrition policy [136, 137].

## Conclusion

Our case study of the adoption of Kenya’s 2012 BMS Act demonstrates that women’s political leadership can counteract the political influence of the transnational formula milk industry and thus help achieve strict national BMS legislation. We show that effective female leadership for BMS legislation can occur in a variety of political offices and positions, including those of ministers and legislators but also those of domestic and international bureaucrats. Importantly, female leaders can leverage their own influence by strategically recruiting male allies and exploiting policy windows.

Future research should continue to investigate the political conditions for the adoption of strict national BMS legislation in line with the International Code. Building on the important literature that demonstrates the pervasive lobbying of the transnational formula milk industry in countries such as Brazil [27], the Philippines [28], Thailand [29] and the United States [138], policy-oriented research should focus more specifically on how governments can overcome this influence in order to adopt and implement strict BMS legislation. Policymakers around the world stand to benefit from such political analyses when developing BMS legislation and defending it against industry opposition [139].

## Electronic supplementary material

Below is the link to the electronic supplementary material.

Supplementary Material 1

Supplementary Material 2

## Data Availability

The data supporting the conclusions of this article are included within the article and its additional files.

## Footnotes

1 It is regularly noted that Kenya was the first country to vote in favor of adopting the International Code at the 1981 World Health Assembly [74, 75], implying that Kenya was a leading proponent of the measure. While Kenya was indeed the first country to cast its vote, this was accidental, as it was randomly selected to commence a roll-call vote in alphabetical order on the resolution [76].

2 This unanimous support of female MPs does not to imply that all women were in complete agreement regarding the BMS bill. For instance, a former chairwoman of the Kenya Nutritionists and Dieticians Institute reportedly advocated against the bill.

3 GAIN’s attempt to stop Kenya’s BMS bill had global repercussions. It resulted in the WHO Executive Board’s decision, in January 2013, to postpone consideration of GAIN’s application to enter into “official relations” with the WHO, requesting that GAIN clarify its “links with the global food industry” and its “support and advocacy of WHO’s nutrition policies, including infant feeding and marketing of complementary foods” [131, 133]. In turn, this likely contributed to GAIN’s severing of ties with Danone in mid-2013 [134].

## Abbreviations

BIG: Breastfeeding Information Group
BMS: Breast Milk Substitutes
GAIN: Global Alliance for Improved Nutrition
HIV: Human Immunodeficiency Virus
IBFAN: International Baby Food Action Network
ICDC: International Code Documentation Centre
International Code: International Code of Marketing of Breast-milk Substitutes
KAM: Kenya Association of Manufacturers
KEBS: Kenya Bureau of Standards
KEWOPA: Kenya Women Parliamentary Association
MIYCN: Maternal Infant and Young Child Nutrition
MoMS: Ministry of Medical Services
MoPHS: Ministry of Public Health and Sanitation
MP: Member of Parliament
MYWO: Maendeleo ya Wanawake Organization
NGO: Non-Governmental Organization
NORAD: Norwegian Agency for Development Co-operation
UNICEF: United Nations Children’s Fund
USAID: United States Agency for International Development
WHA: World Health Assembly
WHO: World Health Organization
WFP: World Food Programme
