# Supplementary material for: Generating political priority for breastfeeding and the adoption of Kenya’s 2012 BMS act: the importance of women’s leadership
Source: Global Health. 2025 May 29;21:32. doi: 10.1186/s12992-025-01127-2 (PMC12123713; doi:10.1186/s12992-025-01127-2)
Supplement: Supplementary file 1 — Supplementary Material 1 [file 12992_2025_1127_MOESM1_ESM.pdf]

Table S1. List of key-informant interviews

#	Institution	Date
1.	Kenyatta University	March 2023
2.	Ministry of Health Kenya	March 2023
3.	Scaling Up Nutrition Civil Society Alliance	March 2023
4.	Ministry of Health Kenya	April 2023
5.	Kenya Women Parliamentary Association	April 2023
6.	University of Nairobi	April 2023
7.	Scaling Up Nutrition Civil Society Alliance	April 2023
8.	Kenya Bureau of Standards	April 2023
9.	Ministry of Health Kenya	June 2023
10.	Kenya Bureau of Standards	October 2023
11.	Kenya Association of Manufacturers	October 2023
12.	Danone	October 2023
13.	Kenya Paediatric Association	October 2023
14.	UNICEF Kenya	November 2023
15.	UNICEF International	March 2024
16.	Ministry of Health Kenya	March 2024
17.	Kenyatta National Hospital	April 2024
18.	Abbott Kenya	April 2024
19.	UNICEF Kenya	May 2024
20.	WHO Kenya	July 2024
21.	IBFAN Kenya	August 2024
