# Supplementary material for: Generating political priority for breastfeeding and the adoption of Kenya’s 2012 BMS act: the importance of women’s leadership
Source: Global Health. 2025 May 29;21:32. doi: 10.1186/s12992-025-01127-2 (PMC12123713; doi:10.1186/s12992-025-01127-2)
Supplement: Supplementary file 2 — Supplementary Material 2 [file 12992_2025_1127_MOESM2_ESM.pdf]

Table S2. Patterns of parliamentary support for Kenya’s 2012 BMS Bill

Member of Parliament	Gender	Political Party	Support for the Bill
Beth Mugo	Female	PNU	+
Cecily Mbarire	Female	PNU	+
Martha Karua	Female	PNU	+
Rachel Shebesh	Female	ODM	+
Joyce Laboso	Female	ODM	+
Millie Odhiambo	Female	ODM	+
Linah Jebii Kilimo	Female	ODM-allied	+
Amos Kimunya	Male	PNU	+
Erastus Mureithi	Male	PNU	+
Njeru Githae	Male	PNU	+
Moses Wetangula	Male	PNU	+
Peter Njuguna Gitau	Male	PNU	+
John Mututho	Male	PNU-allied	+
Abdul Bahari Ali	Male	PNU-allied	+
Phillip Kaloki	Male	PNU-allied	+
Boaz Kipchumba Kaino	Male	ODM	+
Farah Maalim	Male	ODM	+
Charles Keter	Male	ODM	-
Joshua Kutuny	Male	ODM	-
James Rege	Male	ODM	-
Nuh Nassir Abdi	Male	ODM-allied	-
Charles Kilonzo	Male	PNU-allied	-
David Eseli Simiyu	Male	PNU-allied	-
Robert Monda	Male	PNU-allied	-

Note: A “+” indicates support for the proposed bill and against amendments to weaken it during the parliamentary debate on 12th & 19th September 2012 respectively. A “-” indicates opposition to the proposed bill through support for amendments to weaken it. PNU refers to the Party of National Unity, led by Mwai Kibaki. ODM refers to the Orange Democratic Movement, led by Raila Odinga.
